# Integrated Model of Cancer Control for Early Detection and Treatment in Adolescents and Young Adults Living With HIV: Protocol for a Cluster Randomized Controlled Trial

**DOI:** 10.2196/68254

**Published:** 2025-08-29

**Authors:** Sanjana Batabyal, Praveen Zirali, Sonja Hoover, Ronald Mungoni, Nachela Chelwa, Drosin Mulenga, Mildred Lusaka, Laura Nyblade, Madeleine Jones, Catherine Kasongo Mwaba, Michael Mbizvo, Sujha Subramanian

**Affiliations:** 1 Implenomics Dover, DE United States; 2 Population Council Lusaka Zambia; 3 Clinical and Radiation Oncology Society of Zambia (ZASCRO) Lusaka Zambia; 4 RTI International Durham United States

**Keywords:** adolescents and young adults, HIV care, cancer treatment, early detection of cancers

## Abstract

**Background:**

Zambia has one of the highest prevalence rates of HIV among adolescents and young adults (AYA) living with HIV in sub-Saharan Africa, which accounts for half of all new HIV cases as of 2023. Compared to their peers who are not living with HIV, AYA living with HIV are more likely to develop cancer. The most frequently diagnosed cancers among AYA living with HIV in Zambia are cervical cancer, Kaposi sarcoma, and non-Hodgkin lymphoma. Premature cancer mortality among AYA living with HIV is driven by late-stage presentation and poor treatment adherence.

**Objective:**

We aim to develop and test an integrated model of cancer control for AYA living with HIV that can be delivered as an embedded component in existing HIV treatment programs in primary care facilities and linked with specialized treatment at cancer centers.

**Methods:**

We propose a cluster randomized controlled trial to compare the AYAHIV Role-Based Responsibilities for Oncology-Focused Workforce (ARROW) program with a one-time education campaign. The ARROW program consists of interventions at the individual, health care provider, and health system levels. Peer counselors will educate AYA living with HIV through one-on-one and group education sessions and offer care coordination and linkages with clinicians. The HIV and oncology workforce will receive collaborative education and training. The ARROW Health Care Collaborative will connect administrators and policy makers to address system-level barriers. The study will recruit AYA living with HIV between the ages of 15 and 39 years who have been on antiretroviral therapy for at least 6 months and are not pregnant; the cancer treatment cohort will enroll AYA living with HIV who have been diagnosed with cervical cancer, Kaposi sarcoma, or non-Hodgkin lymphoma in Lusaka, Zambia. Half of the 18 HIV facilities have been randomly assigned to a one-time educational campaign and the other half to the ARROW intervention. Participants in the cancer treatment cohort will be randomized into 1 of the 2 study arms. We will conduct economic evaluations to assess the cost-effectiveness of the ARROW program. We will use an intent-to-treat approach to test the hypothesis that AYA living with HIV in the ARROW program will have higher uptake of diagnostic services, increased adherence to treatment, and improved outcomes compared to those receiving one-time education.

**Results:**

As of March 2025, early detection cohort recruitment concluded with 3442 participants and cancer treatment cohort enrollment is ongoing, with 105 participants thus far. Results pertaining to the 12-month end points will be available in early 2026.

**Conclusions:**

If successful, the ARROW program will offer a model to improve cancer prevention, early diagnosis, and treatment through improved integration between HIV and cancer services. Furthermore, ARROW can provide a framework for implementing expanded services, such as survivorship care, for AYA living with HIV.

**International Registered Report Identifier (IRRID):**

DERR1-10.2196/68254

## Introduction

### Background

Adolescents and young adults (AYA), those aged 15 to 39 years, represent a growing share of people living with HIV worldwide. Zambia has one of the highest prevalence rates of HIV among AYA in sub-Saharan Africa; as of 2023, there are approximately 90,000 AYA between the ages of 10 and 19 years living with HIV, with only half of that population receiving antiretroviral therapy (ART) [1]. The AYA population also accounts for half of all new recorded infections in the country [1]. Compared to their peers who are living without HIV, AYA living with HIV are at an increased risk of developing cancer [2-4]. Cancers most frequently diagnosed among AYA living with HIV in Zambia are Kaposi sarcoma (KS), non-Hodgkin lymphoma (NHL), and cervical cancer (CC) [5-9]. The incidence of these cancers increases with a lower cluster of differentiation 4 cell count (CD4); therefore, adherence to ART is paramount [10]. Unfortunately, AYA living with HIV are among the most vulnerable groups with poor adherence to ART, and there is no large-scale cancer control program to facilitate early diagnosis and completion of guideline-recommended treatments in Zambia [11,12].

A cancer program focused on AYA living with HIV and tailored to the Zambian limited resource setting is needed to reduce premature mortality. Data on malignancies within this group are sparse, but late-stage presentation, unplanned breaks from cancer treatment, and abandonment of treatment have been reported among young patients with cancer in resource-limited settings [13-15]. These factors result in high morbidity and premature mortality among AYA living with HIV and diagnosed with cancer. Late-stage presentation and lack of treatment adherence are key drivers of premature cancer mortality among AYA living with HIV in Zambia.

AYA living with HIV are a vulnerable population who face psychosocial issues, stigma, and comorbidities that affect their health care-seeking behavior [16]. In Zambia, young patients living with both HIV and cancer have unmet needs related to information about health risks, coping strategies for medication side effects, positive peer and family support, and activation of personal agency [17-20]. Furthermore, during the transition from pediatric to adult HIV clinics, many of these individuals are lost to care [21-25]. Health care providers who specialize in HIV care and oncology face barriers, such as time constraints, with regard to treating cancer in AYA living with HIV, which inhibits their ability to deliver optimal care [26,27]. There are also no linkages for collaborative management of cancer in AYA living with HIV by primary care providers specializing in HIV care and cancer specialists. At the health system level, some key barriers include weak referral systems and a lack of standardized protocols to implement AYA-friendly care [28-32].

The Zambian guidelines recommend CC screening for all female AYA living with HIV who are sexually active, regardless of age [33]. However, screening among this population is low, despite the availability of free health care services. According to recent studies [18,34], <40% of AYA living with HIV have undergone screening for CC. There are no screening tests available for KS or NHL, but increasing awareness of the risk factors and symptoms, along with physical examinations, could reduce late presentation [13,29]. In fact, strategies to facilitate early detection of KS have shown promise in other African countries. A recent study in Uganda [35] used education messaging (comic strip and video) to increase KS awareness and knowledge. Furthermore, researchers in Zimbabwe developed a physical examination checklist to identify symptoms of KS [36]. The Cancer Diseases Hospital, which is the main referral center for treatments in Zambia, has adopted the National Comprehensive Cancer Network’s (NCCN’s) Harmonized Guidelines for sub-Saharan Africa for AYA oncology to optimize treatment. The NCCN guidelines will be most effective when paired in tandem with adequate treatment adherence support for AYA living with HIV.

AYA-focused programs have been successful in other settings, especially in high-income countries. These programs use a variety of models to promote collaboration across multidisciplinary teams and integrate medical and psychosocial care for AYA [37-39]. In sub-Saharan African countries such as Zambia, multiple barriers need to be addressed to facilitate early diagnosis in the primary care setting and to ensure receipt of full treatment course in cancer centers [40].

### Objective

We aim to develop and test an integrated model of cancer control for AYA living with HIV that can be delivered as an embedded component in existing HIV treatment programs in primary care facilities and linked with specialized treatment in cancer centers.

## Methods

### Overview and Conceptual Framework

#### Overall Approach

We propose a program that embeds cancer control strategies for AYA living with HIV into existing HIV treatment programs in primary care facilities and at cancer centers in Zambia by strengthening and supporting the existing clinical teams to optimize cancer care delivery. We used theory-informed multilevel strategies to create the AYAHIV Role-Based Responsibilities for Oncology-Focused Workforce (ARROW) program to increase uptake of services for early diagnosis and improve compliance with cancer treatment for CC, KS, and NHL. ARROW will use an evidence-driven approach to select low-cost, multilevel peer-to-peer support and learning strategies to address identified barriers and delineate roles played by HIV and cancer care providers to deliver guideline-directed care for early diagnosis and cancer treatment. Each of the proposed strategies has been tested in the Zambian and sub-Saharan African context and shown to be feasible [18,41-45].

**Figure 1:**
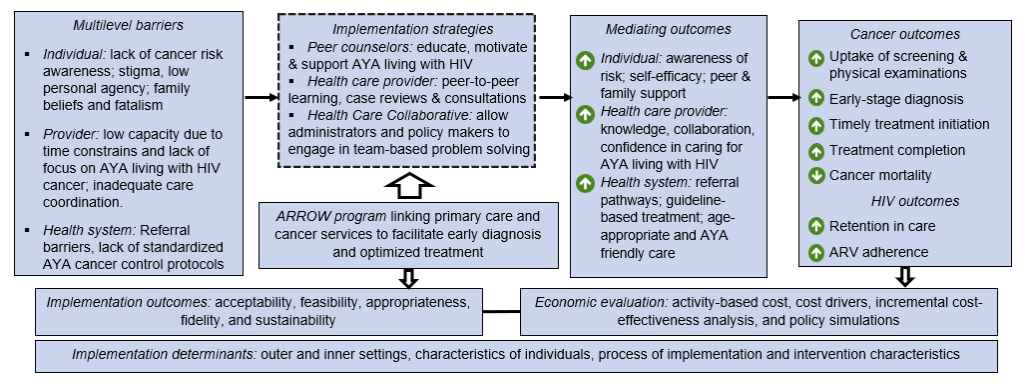
Conceptual framework for addressing barriers to reduce cancer mortality. ARROW: AYAHIV Role-Based Responsibilities for Oncology-Focused Workforce; ART: antiretroviral therapy; AYA: adolescents and young adults.

Our overall approach is based on the evidence-based strategy of peer support for engagement and learning [46-54]. Figure 1 presents the theoretical framework, which is based on the socioecological model [55]. We have created the ARROW program with strategies that focus on barriers at the individual, health care provider, and health system levels. These strategies are guided by the capability, opportunity, motivation, and behavior model, in which capability (eg, knowledge and cancer risk), opportunity (eg, availability of high-quality screening and treatments), and motivation (eg, self-efficacy and care-seeking behavior) interact to facilitate behavior change [55].

#### Interventions

The ARROW program is a holistic intervention that is meant to support HIV and cancer care at 3 different levels: individual, health care provider, and health system.

#### Individual Level

There is growing evidence on the role that peer support can play in addressing individual and interpersonal barriers to improve knowledge and encourage AYA to seek medical care [56-62]. We will place trained peer counselors (PCs) in HIV treatment facilities and at the Cancer Diseases Hospital to educate AYA living with HIV, provide emotional support, and offer care coordination and linkages with the clinical team. PCs will offer support for early diagnosis of cancers, completion of cancer treatment, and ART adherence as required. They will coordinate care with health care providers and serve as an extension of the clinical team by conducting physical examinations to identify signs of KS and NHL and offering coping strategies for ART and chemotherapy side effects.

A total of 21 PCs, 10 (48%) males and 11 (52%) females between the ages of 20 and 39 years, will be recruited. Of the 21 PCs, 18 (86%; 9/18, 50% males and 9/18, 50% females) will receive traning for placement in HIV treatment centers. The remaining 3 PCs (14%; 1/3, 33% male and 2/3, 67% females) will be posted to the Cancer Diseases Hospital. The recruitment of more female PCs is due to the heavy burden that CC places on the health care system. Our existing 3-week training curriculum, from a previous study [18] conducted by the research team, will be tailored for PCs and will include training on appointment tracking systems. The curriculum will include standardized operating procedures for implementing the group education sessions to include additional details on cancer risk, early diagnosis approaches, and optimal treatments. Those assigned to the Cancer Diseases Hospital will receive training on how to help AYA living with HIV manage treatment side effects and assist with their adherence to ART while undergoing cancer treatment. The PCs will conduct site visits of the HIV facilities and the Cancer Diseases Hospital to become familiar with the staff, the layout of the clinics, and the services available.

Table 1 summarizes the 4 core PC roles and their corresponding activities. The PCs will offer 6 group education sessions at each ARROW HIV facility per month (one module each month) for the first 12 months, along with one-on-one education. For the remaining follow-up period, PCs will offer one-on-one education as needed. All other activities will continue throughout the trial.

**Table 1:** Key functions of peer counselors (PCs).

Functions	HIV treatment facility	Cancer diseases hospital
HIV and cancer knowledge	Group and one-on-one education on cancer early diagnosis and ART^a^ adherence	One-on-one education on cancer treatment and ART adherence; some group sessions
Social and emotional support	Group sessions and individual meetings to discuss coping with stigma, family stressors, and side effects (ART or cancer treatment)	Group sessions and individual meetings to discuss coping with stigma, family stressors, and side effects (ART or cancer treatment)
Care-seeking behavior	Motivational interviews; PCs offer convenient times for physical examinations; reminder phone calls before visits	Documenting treatment plan; tracking of visits to remind AYA^b^ living HIV about visits; identifying and addressing barriers
Care coordination	Accompany AYA living with HIV to referral visits; facilitate delivery of results to the HIV care team; inform AYA living HIV about the next steps	Ensure that the cancer care team is aware of any barriers to adherence; facilitate communication with the HIV care team

^a^ART: antiretroviral therapy.

^b^AYA: adolescents and young adults.

Educational materials will be made for the 2 study arms of the randomized controlled trial by adapting existing group and one-on-one educational materials, such as training modules previously tested for KS awareness or the NCCN AYA Guidelines for Patients. These materials will be presented at pretesting workshops. Trained facilitators will present materials to ensure that they are age-appropriate by assessing participants’ ability to understand, whether the topics and contents address key issues faced by the age group, and whether AYA living with HIV can embrace and follow the solutions or advice offered. Using the feedback received, we will finalize the modules and brochure. All adapted education materials for AYA living with HIV will be available in English, Bemba, Nyanja, and Tonga. Health care provider guidance documents will be available in English, as in previous projects; all Zambian health care providers are fluent in English.

A treatment plan based on the NCCN treatment and survivorship plans will be developed for study participants at the Cancer Diseases Hospital. The PCs will update treatment plans for AYA living with HIV through the treatment course, which will serve as a comprehensive record that can also be placed in the patient file at the HIV treatment center that the AYA living with HIV will return to after treatment completion.

#### Health Care Provider Level

Team-based care is critical for optimal oncology care delivery [61,62]. Formalized communication channels, collective learning, and clear team roles are the cornerstone of collaborative care teams [63,64]. The ARROW Provider Network will use a tailored Project Extension for Community Healthcare Outcomes approach to link pediatric and adult health care providers who specialize in HIV care in the primary care facilities with counterparts at the Cancer Diseases Hospital to enable peer-to-peer learning, review of patient cases, and expert consultations [44].

Guidance documents and brochures will be created for health care providers by inviting a mix of pediatric and adult care providers to cocreating workshops. These materials will contain background information, AYA-friendly care delivery, age-appropriate engagement with AYA living with HIV, monitoring of ART adherence, flowcharts with referral pathways for early diagnosis, process steps to implement guideline-recommended cancer treatment for AYA living with HIV, documentation of services provided (eg, in the SmartCare electronic health record, which is used in HIV facilities and capture visit details, medications, and laboratory tests), and a charter with roles and responsibilities for clinical teams in the HIV treatment centers and those at the Cancer Diseases Hospital. Facilitators will use the NCCN sub-Saharan African harmonized guidelines for each cancer, along with the AYA guidelines, and create guidance documents focused on AYA living with HIV that are tailored to the Zambian setting.

Health care providers will also receive in-person training at each of the 9 HIV facilities selected to implement the ARROW program and at the Cancer Diseases Hospital before initiating the implementation trial. These training programs will also include sessions on understanding stigma faced by AYA living with HIV, delivering age-appropriate and AYA-friendly care, and best practices for communication with this population using previous training tools [17]. All HIV care providers (both pediatric and adult) will be encouraged to attend a session. We will hold additional training as needed, including refresher training sessions during the 36-month trial.

We will initiate activities to facilitate collaborative learning and coordination by hosting (1) monthly peer-to-peer web-based sessions with presentations from Zambian and international physicians working to improve AYA cancer care, case reviews for AYA living with HIV, and expert consultation panels to address specific questions from the ARROW Provider Network; (2) an annual in-person meeting in Lusaka to review progress and next steps; and (3) an in-facility biannual seminar with adult and pediatric teams on specific issues related to AYA living with HIV.

#### Health System Level

We will initiate the ARROW Health Care Collaborative, which will bring together health system administrators and Zambian Ministry of Health policy makers to foster team-based problem solving and implementation of solutions to address system-level barriers (eg, barriers related to referrals). We will also use a user-centered design process to cocreate education materials and guidance documents to implement ARROW.

We will invite 6 health care administrators from Lusaka province and 4 Ministry of Health policy makers to join the Collaborative to foster team-based problem solving. These individuals will meet quarterly to review implementation challenges related to referral pathways and other system-level barriers to receiving services to facilitate early diagnosis and optimal cancer treatment. Each meeting will end with clear action steps toward resolving the barriers and procedures that will be used to monitor the implementation of the proposed solutions. The first meeting of the Collaborative will take place 6 months before the start of the trials to allow adequate time to identify and address any gaps identified through the readiness assessment. The reduction in barriers will benefit not only the ARROW program participants but also all AYA living HIV in Lusaka province.

### Randomized Controlled Trials

#### Overall Study Design

##### Overview

We will compare the ARROW program to a one-time education campaign that gives informational brochures to AYA living with HIV and HIV care providers. Given the acute need for cancer control strategies for AYA living with HIV to reduce premature mortality, planning consultations with community stakeholders deemed it unethical to use “usual care” as the comparison arm. We will, therefore, test the hypothesis that the ARROW program will be more effective than a one-time education campaign (education brochures to AYA living with HIV and HIV providers) in increasing care received by AYA living with HIV to facilitate early diagnosis (physical examination for KS and NHL, CC screening, and timely referrals) and in improving adherence to treatment among AYA living with HIV diagnosed with cancer. We will also assess implementation outcomes (based on the Proctor framework), analyze determinants of implementation (based on the Consolidated Framework for Implementation Research), and conduct economic evaluation of the ARROW program [65,66].

##### Early Detection Cohort

We propose a cluster randomized study to be implemented at 18 HIV treatment facilities in Lusaka province. Three facilities serving each type of regional population (urban, periurban, or rural populations) will be randomly assigned to a study arm: the ARROW program or one-time education. Overall, 9 (50%) facilities will be assigned to each arm of the trial, with data collection at baseline and at 12-, 24-, and 36-month follow-up. At each of the 18 clinics, we will enroll 100 men and 100 females resulting in a total of 3600 participants in the early detection cohort. The 200 AYA living with HIV per facility will be selected by first reviewing the HIV register and identifying those who meet the study inclusion criteria: 15 to 39 years of age, on ART for at least 6 months, not pregnant, and with no pending plans to move from the current residence during the 3-year study duration. For all the 3600 participants, we will collect baseline data and capture information on study end points from the SmartCare system, and the supplemental data will be collected by PCs and trained study data collectors (eg, tracking referrals for all participants and obtaining results of biopsies).

##### Cancer Treatment Cohort

This will take place entirely at the Cancer Diseases Hospital, with some patients being recruited from the University Teaching Hospital. A total of 500 AYA living with HIV who have been diagnosed with CC, NHL, or KS will be recruited into the study, with 50% (250/500) of the patients in each trial arm (ie, one-time educational campaign vs the ARROW intervention). Patients will be surveyed annually at 12, 24, and 36 months. Trained data collectors will obtain consent and enroll AYA living with HIV, aged 15 to 39 years. We will be aiming for the following targets for each cancer type: 50% (250/500) of the participants with CC, 30% (150/500) of the participants with KS, and 20% (100/500) participants with NHL (with an overall equal number of male and female AYA living with HIV for KS and NHL). While AYA living with HIV who are pregnant will be excluded, as they require specialized care, those who become pregnant during the trial period will remain enrolled in the study; CC screening and physical examinations for KS and NHL will be offered based on Zambian guidelines pertaining to pregnancy.

**Figure 2:**
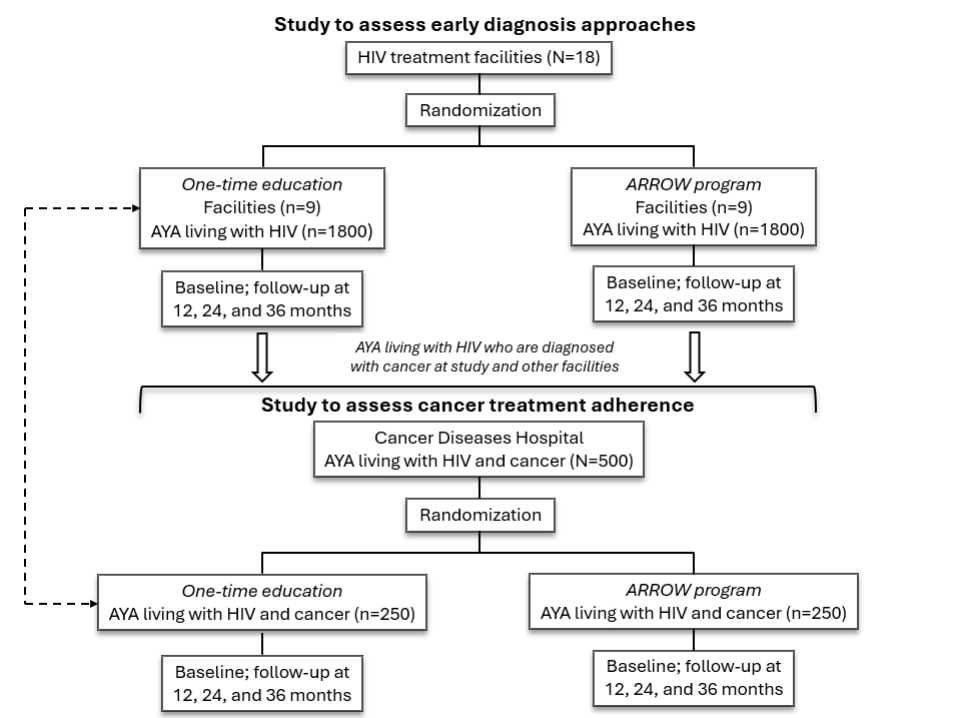
Experimental design and sample sizes. ARROW: AYAHIV Role-Based Responsibilities for Oncology-Focused Workforce; AYA: adolescents and young adults.

We hypothesize that, by facilitating peer support to address barriers and gaps at multiple levels, the ARROW program will be more effective than the one-time education campaign, but more expensive as well. As shown in Figure 2, we will simultaneously conduct separate randomized controlled trials at the HIV treatment facilities and at the Cancer Diseases Hospital. The trials will be initiated upon completion of the preimplementation preparation activities. We will then embed PCs into the care teams at the HIV facilities and the Cancer Diseases Hospital and initiate the planned implementation activities of the ARROW Provider Network in the ARROW program arm. The comparison arm will receive the one-time education campaign: health care providers at the HIV facilities (particularly physicians and nurses) will receive a brochure with key guidance for early diagnosis of cancers in AYA living with HIV, and the AYA living with HIV upon recruitment will also receive education brochures at both the HIV facilities and the Cancer Diseases Hospital. The Health Care Collaborative will continue to meet as planned on a quarterly basis.

#### Study End Points

##### Early Detection Cohort

We will assess effectiveness based on dual primary end points. The first primary end point is the proportion of those who complete a physical examination visit for KS and NHL and the proportion of those who are up to date with CC screening (female participants only) at 12 months. The second primary end point is the proportion of those who complete the recommended follow-up supplemental or diagnostic procedures (within 3 months of receiving follow-up referral), those who initiate cancer treatment (within 3 months of diagnosis), and those who complete additional cancer detection visits over 36 months. The secondary end point, evaluated at 36 months, is the proportion of those diagnosed at an early stage (among those diagnosed with KS, NHL, and CC). We will likely have a few cancer cases in the study facilities. Therefore, these findings will not be conclusive but will offer insights into the potential impact of the ARROW program on facilitating diagnosis at an early stage.

##### Cancer Treatment Cohort

The primary end point is the proportion of those who are adherent to cancer treatment (complete all treatment modalities as prescribed; 2-week delays will be allowed) at 12 months after diagnosis, the secondary end point is the proportion alive at 12 months after diagnosis, and the tertiary end point is the proportion with recurrence at 24 and 36 months (among those with 24- and 36-month follow-up).

For all the AYA living with HIV, we will report adherence to ART (proportion of those filling prescriptions at least every 3 to 6 months) and viral load at 12, 24, and 36 months. Viral load testing is generally conducted at least annually for AYA living with HIV as part of routine guideline-recommended practice. We will reinforce the need for regular viral load testing as part of health care provider training and have set aside funds to conduct viral load testing for study participants for whom this information is unavailable in the medical record.

#### Sample Size and Recruitment

##### Early Detection Cohort

We will use stratified random sampling to select an equal number of male and female participants and a mix of AYA living with HIV with perinatally and behaviorally acquired HIV. In terms of age distribution, 20% (720/3600) of the sample will be aged 15 to 19 years, 20% (720/3600) will be aged 20 to 24 years, and 60% (2160/3600) will be aged 25 to 39 years. We will oversample AYA living with HIV in each stratum to account for AYA living with HIV who may not meet inclusion criteria or who decline to participate. HIV facility staff will contact AYA living with HIV or consenting adults (for those aged 15 to 17 years) to discuss the study, and those interested will meet with trained data collectors who will assess eligibility and obtain consent or assent. The sample size was calculated assuming conventional specifications (α=.025 for each end point, 2-sided tests) based on the dual primary end points and allowing for the detection of an absolute 20% point improvement in the primary end points, which is our minimum threshold to consider the ARROW program successful. For the one-time education arm, given the current low level of uptake of early diagnosis services, we estimate uptake of physical examinations to be about 15% (15/100) and CC screening to be about 20% (20/100) at 12 months. With our proposed sample size of 3600, we will be able to detect a 20% difference in uptake of physician examination (35% in the ARROW arm) with over 90% power and CC screening (40% in the ARROW arm) with over 85% power. We can achieve this even after accounting for up to 10% attrition at 12 months (a previous study conducted by the research team achieved this retention rate without the level of active participant contact planned for this study) [67]. We estimate that the interclass correlation coefficient will be in the range of 0.03 to 0.05 because correlation coefficients for binary data are typically quite small, especially when physical examinations and screening uptake are very low [68].

##### Cancer Treatment Cohort

We will use stratified random assignments to place patients in each arm of the trial. Once we have enrolled 10 patients with each type of cancer, we will randomly assign half to the ARROW program and the other half to the one-time education arm. All patients will have a minimum of 12 months of follow-up, and some will have up to 36 months of follow-up. Data will be collected at baseline and then annually for the 3-year duration of the trial from the AYA living with HIV through surveys and by abstracting information from the Cancer Diseases Hospital records. The sample size (n=500) was calculated using conventional specifications (α=.05; 2-sided tests) based on the primary end point and allowing for an absolute 15% point improvement; the estimated current level of adherence is 75%, which will increase to at least 90% with the ARROW program. With 50% (250/500) of the patients in each arm, we will have more than 90% power to detect this difference.

### Ethical Considerations

This study was approved by the institutional review boards at the Population Council (Protocol 1019) and the ERES (Excellence in Research Ethics and Science) Converge (Ref. No. 2022-Dec-003) and has been registered on ClinicalTrials.gov (NCT06004011). For the AYA living with HIV aged <18 years, we will obtain both written parental consent and assent from the minors. The AYA living with HIV aged ≥18 years will be asked to provide written informed consent. Trained data collectors will administer and document informed consent. Data will be collected via a secure data platform, and no identifiable data will be collected in the baseline and annual surveys. The participants will not receive monetary compensation for participation in study education sessions and other activities. The participants will receive ZK 100 (US $4.23) for completing the data collection at baseline and at annual touchpoints. This study also has a data safety monitoring board that provides ongoing evaluation of the study’s progress. This board has the authority to review any adverse events and provide guidance for any protocol modifications or termination of the study. The board comprises 5 members from nongovernmental organizations and the Zambian Ministry of Health. Any protocol changes, or other modifications, with be shared with the institutional review boards and the data safety monitoring board.

### Data Collection

In the HIV treatment facilities, we will generate electronic data files for the study cohort, and in other settings, such as referral facilities and the Cancer Diseases Hospital, the study team will abstract data from electronic databases or paper files. We will also conduct surveys to collect data from the AYA living with HIV and health care providers. The PCs will document their interaction with the AYA living HIV in the PC tracking tool, and the health care provider participation in the ARROW Provider Network will be captured in the study database. These details will be used to determine the “dose” of the ARROW program. All data will be collected by trained data collectors, who will undergo continual monitoring, and study coordinators, who will confirm fidelity to protocol specifications by random in-person review. Refer to Table 2 for the end point definitions and the biological, self-report, and clinical records data collected at baseline and at 12-, 24-, and 36-month follow-ups.

**Table 2:** Data collection at baseline and at 12-, 24-, and 36-month follow-ups.

Constructs and measures		Instrument and specification	Source	Timing
**Data elements for primary, secondary, and tertiary end points: HIV and cancer care continuum**				
	Proportion of participants completing physical examination (KS^a^ and NHL^b^) and up to date with CC^c^ screening	Confirmation of services will be based on clinical records and PC^d^ tracking tool (primary end point at 24 mo)	Biological, imaging, and clinical procedures identified through clinical records and study database	12, 24, and 36 mo
	Proportion of participants completing diagnostic tests and initiating treatment in ≤90 d	Measured as days from referral for testing and cancer diagnosis, respectively; based on chart review and study data	Biological, imaging, and clinical procedures identified through clinical records and study database	24 and 36 mo
	Proportion of participants completing repeat examinations and screening at the recommended interval	Screening every 2 y for CC and annual examination (especially when CD4^e^<100) based on the Zambian guidelines	Biological, imaging, and clinical procedures identified through clinical records and study database	36 mo
	Proportion of participants with early-stage diagnosis^f^	Stage 1 or 2 for NHL and CC; good risk for KS^g^	Biological, imaging, and clinical procedures identified through clinical records and study database	36 mo
	Proportion of participants adherent to cancer treatment^f^	Complete cancer treatment modalities as recommended	Biological, imaging, and clinical procedures identified through clinical records and study database	12 mo after diagnosis
	Proportion of participants alive at 12 mo after diagnosis^f^	Follow-up conducted by PCs and entered in study database	Biological, imaging, and clinical procedures identified through clinical records and study database	12 mo after diagnosis
	Proportion of participants with recurrent disease^f^	AYA^h^ living with HIV and cancer who experience cancer recurrence	Biological, imaging, and clinical procedures identified through clinical records and study database	36 mo
	Proportion of participants adherent to ART^i^ and viral load	Prescription filled every 3 to 6 mo; blood test	Biological, imaging, and clinical procedures identified through clinical records and study database	Annually
**HIV status, demographics, mediating outcomes, intervention dose, and clinical services**				
	HIV status and diagnosis date	Perinatally or behaviorally infected; CD4 count	Clinic record	Baseline
	Demographics and socioeconomic status	Research Triangle Institute AYA Zambia survey	Self-report	Baseline
	Self-efficacy (AYA living with HIV)	Generalized self-efficacy Scale [69]	Self-report	Baseline and 12, 24, and 36 mo
	Social support and mental health (AYA living with HIV)	Brief social support and patient health questionnaires [69,70]	Self-report	Baseline and 12, 24, and 36 mo
	HIV and cancer stigma (AYA living with HIV)	Anticipated stigma measures [71,72]	Self-report	Baseline and 12, 24, and 36 mo
	Cancer risk (AYA living with HIV)	Updated Research Triangle Institute cancer survey	Self-report	Baseline and 12, 24, and 36 mo
	Group education and sessions (AYA living with HIV)	Number and proportion of education sessions attended (dose)	PC tool; study database	12, 24, and 36 mo
	One-on-one education (AYA living with HIV)	Number of women receiving each level of strategy (dose)	PC tool; study database	12, 24, and 36 mo
	HIV and cancer team training (health care provider)	Proportion who attended initial training sessions (dose)	PC tool; study database	12, 24, and 36 mo
	Network meetings (health care provider)	Number and proportion of meetings attended (dose)	PC tool; study database	12, 24, and 36 mo
	Clinical team knowledge, confidence in ability, and collaboration (health care providers)	Survey on skills and patient communication; stigma related to AYA living with HIV (modified Nyblade instrument [73]) and peer learning and coordination	Self-report	Baseline and annually
	Diagnostic procedures by cancer type	Number of supplemental and diagnostic tests conducted	Clinical record	12, 24, and 36 mo
	Treatment modality by cancer type	Chemotherapy sessions; surgery; type of radiation	Clinical record	12, 24, and 36 mo

^a^KS: Kaposi sarcoma.

^b^NHL: non-Hodgkin lymphoma.

^c^CC: cervical cancer.

^d^PC: peer counselor.

^e^CD4: cluster of differentiation 4.

^f^Among AYA living with HIV and diagnosed with cancer.

^g^NHL stage based on the Lugano classification, CC stage based on the International Federation of Gynecology and Obstetrics, and KS stage based on the AIDS Clinical Trial Group system.

^h^AYA: adolescents and young adults.

^i^ART: antiretroviral therapy.

### Analysis of Hypotheses

We will use an intent-to-treat approach to test the hypotheses (related to the dual primary and secondary end points) that AYA living HIV in the ARROW program, compared with those receiving one-time education, will have higher uptake of early diagnostic services, increased adherence to treatment, and improved outcomes.

For the facilities assigned to the intervention group in the early detection cohort, we will examine the effects at the individual level across the 2 randomization groups. Given the need to consider the influence of the cluster randomization, we will use generalized estimating equation models to estimate the effect across the trial arms. If the Hausman assumption of correlation between the random and fixed effects is violated, then we will include fixed effects representing cluster identification [74]. For the cancer treatment cohort, we will generate summary statistics for the end points and test statistical significance. We will also conduct logistic regressions to identify key factors that predict adherence.

For both trials, we will adjust for baseline covariates, including sociodemographic factors, should the initial descriptive analyses suggest differences in the distribution of these factors across the study arms. We will also explore the use of propensity scores to control for systematic differences between the AYA living with HIV in the 2 trial arms. In further analyses, we will compare differences in mediating outcomes across the randomized groups and examine the potential moderating roles of key individual behavioral and social factors hypothesized to influence adherence along the cancer care continuum (eg, social support, stigma, and self-efficacy), as shown in Figure 1. We will test the “dose” of the ARROW program received as a covariate in these models. In additional analysis, we will explore differences in the end points by age groups and type of cancer, as appropriate. We will also assess whether knowledge, self-efficacy, social support, and stigma differ by age groups and among AYA living HIV, both with and without cancer.

For measures where we have baseline and 12-, 24-, and 36-month data (eg, self-efficacy and stigma), we will perform difference-in-difference analysis, which will allow for comparisons over time and across the study arms. To understand potential variation across the HIV treatment facilities, we will conduct comparisons to analyze differences in mediating outcomes for the AYA living with HIV and the study end points. Furthermore, to understand ARROW program’s impact on health care providers, we will assess dose of the intervention (network session attendance) and change in knowledge, confidence in ability to care for AYA living with HIV, and peer collaboration.

### Implementation Outcomes

For assessing the ARROW program, we will collect measures (as outlined in Table 3) from those receiving and implementing ARROW: 250 selected AYA living with HIV with follow-up data collection at the HIV treatment clinics and 250 AYA living with HIV at the Cancer Diseases Hospital, 36 HIV care providers, 12 oncology staff from the Cancer Diseases Hospital, 10 Health Care Collaborative members, and all the PCs. We will collect similar measures focused on the education campaign from those receiving the brochures in the one-time education campaign arm: 250 AYA living with HIV in the early detection cohort, 250 AYA living with HIV in the cancer treatment cohort, and 36 HIV care providers.

**Table 3:** Implementation outcomes measurement.

Constructs	Measures	Stakeholder and data	Frequency
Acceptability, feasibility, and appropriateness	AIM^a^, FIM^b^, and IAM^c^ 4-item measures	AYA^d^ living with HIV, PCs^e^, and health care provider (HIV and cancer) surveys	12-, 24-, and 36-mo follow-up
Fidelity (group and one-on-one education, health care provider meetings, and education campaign)	All content is delivered, or brochures are provided; meetings take place at the frequency and sequence planned	PC tracking tools, the ARROW Provider Network, and the Health Care Collaborative meeting minutes	12, 24, and 36 mo
Sustainability	SMSS^f^ subscales specific to strategies	PCs, health care providers, and administrators	12 and 36 mo

^a^AIM: Acceptability of Intervention Measure.

^b^FIM: Feasibility of Intervention Measure.

^c^IAM: Intervention Appropriateness Measure.

^d^AYA: adolescents and young adults.

^e^PC: peer counselor.

^f^SMSS: Sustainment Measurement System Scale.

Our measurement will include the 4-item questions from the Acceptability of Intervention Measure, the Feasibility of Intervention Measure, and the Intervention Appropriateness Measure [75]. Fidelity will be assessed using data collected in the PC tracking tool and Network meeting. The fidelity measures described in Table 3 will be compiled annually, and summary statistics (eg, proportion of sessions in which all content was delivered) will be generated to assess compliance with study protocols. Sustainability will be assessed using subscales of the Sustainment Measurement System Scale [76]. We will review the scores and fidelity measures and compare scores available for both the ARROW and one-time education arms to assess implementation success and draw lessons to improve and optimize the ARROW program.

We plan to conduct interviews and focus groups to identify the underlying moderating factors associated with the successful implementation of strategies, along with facilitators and barriers to inform future scale-up activities. At 12 months, trained data collectors will interview PCs and health care providers as described for the quantitative measures. We will interview this group to seek feedback on their level of preparedness to implement the strategies, their assessment of the quality of the delivery, the level of responsiveness of the AYA living with HIV, whether they felt there were facilitators to deliver the strategies and potential barriers, and suggestions to address the barriers. We will also interview the AYA living with HIV to seek feedback on the ARROW program and one-time education campaign.

At 36 months, at the end of the trials, we will conduct focus groups with PCs, AYA living with HIV, HIV care providers, and oncology care providers. These focus groups (7-10 participants) will explore topics related to the sustainability of the ARROW program and the one-time education campaign, drawing on the findings from the results of the Sustainment Measurement System Scale survey. We will critically discuss interpretations of the data until they reach a consensus on the dominant themes and meanings. The findings will offer insights to improve the ARROW program and understand perceptions of the one-time education campaign.

### Cost-Effectiveness and Return-on-Investment Scenarios

We will assess the incremental cost-effectiveness of the ARROW program compared with the one-time education campaign. As shown in Table 4, we will generate the cost of start-up activities to understand the resources required to plan future implementation, the activity-based cost of strategies during the intervention trials, and the estimated cost during scale-up. We will use a previously validated instrument, the Cost Assessment Tool, to collect resource use and cost information [77].

**Table 4:** Cost data collection and analysis by phase.

Phase and data category		Purpose	Data elements (examples)
**Before implementation**			
	Start-up costs	To assess the cost of planning to implement strategies	Formalizing plans for the intervention; hiring staff; conducting training, readiness assessment, and strengthening processes
**Intervention implementation (randomized controlled trials)**			
	Implementation of activity-based cost data	To assess the cost of implementing and maintaining interventions	Implementation activities: delivering strategies, tracking outcomes, quality assurance, data collection, and evaluation Patient cost: travel cost and time lost from work
	Resource use	To standardize cost	Labor hours (peer counselors and project staff) and nonlabor resources
**Future expansion**			
	Scale-up cost	To estimate the cost of large-scale implementation	Fixed versus variable activity-based cost estimates of implementation strategies to project economies of scale

Our main goal is to estimate the implementation cost from the program perspective. We will estimate labor hours by prospectively tracking time spent by each project staff member, including PCs, on a predefined set of activities (individuals will report their time monthly) and use hourly wages to calculate cost. We will capture time spent by health care providers, administrators, and policy makers participating in the ARROW Network and Collaborative using from the meetings. We will also document the expenditures on nonlabor resources to produce education materials, hosting group education sessions, the ARROW Provider Network meetings, and the cost of providing travel support. Using activity-based costing, we will allocate separate costs for supporting early diagnosis services and treatment adherence.

On the basis of these cost estimates and the primary end points from the trial, we will conduct incremental cost-effectiveness analysis by generating the cost per AYA living with HIV undergoing early detection services (physical examinations for KS and NHL and screening for CC) and the cost per AYA living with HIV and cancer completing recommended treatment modalities. Using standard economics methodology, we will explore economies of scale related to the implementation costs that can be achieved during scale-up [77,78]. We will also assess the key drivers of the cost of the ARROW program.

Using the trial data on estimated shift in stage at diagnosis and treatment adherence rates, along with supplemental information on prevalence of KS, NHL, and CC and mortality by stage at diagnosis, we will estimate the projected difference in mortality between ARROW and one-time education for AYA living HIV in Zambia. For CC screening, we will also estimate the incremental change in mortality using a validated microsimulation model, as screening not only will identify CC at an early stage but also can prevent CC by treating precancerous lesions [79,80].

We will derive the cost per life year saved (for all the 3 cancers combined), and our estimates will include both direct and indirect costs (patient time loss). The latter represents the opportunity cost of engaging in health services. We will assess whether the derived costs per life year saved are cost-effective or considered acceptable for implementation based on cost-effectiveness profiles of other strategies or interventions already implemented [81]. The estimated cost and effectiveness will be used to assess return on investment based on resources required to implement the ARROW program and the economic burden that will be reduced by avoiding premature mortality and treatments for adverse events related to KS, NHL, and CC for AYA living with HIV.

Furthermore, we will conduct policy simulations to assess the impact of scaling up ARROW and perform a sensitivity analysis, varying the range of effectiveness and cost estimates, and generate potential best- and worst-case projections. We will create tornado and spider diagrams to display this uncertainty graphically to policy makers. This cost-effectiveness analysis will be complemented by a budget analysis, which will identify the financial outlays that will be required annually to implement the ARROW program at scale.

## Results

Recruitment for both the early detection cohort and the cancer treatment cohort began in June 2024. Recruitment for the early detection cohort concluded in February 2025, and we have enrolled a total of 3442 individuals. Of these 3442 participants, 1731 (50.29%) were enrolled in the 9 facilities randomized to the intervention arm and 1711 (49.74%) were enrolled in the 9 facilities in the control arm. The ARROW intervention was launched at all intervention facilities by December 2024. Recruitment for the cancer treatment cohort began in July 2024 and is ongoing; as of March 2025, 105 individuals have been enrolled. Of these 105 participants, 52 (49.5%) are receiving the one-time educational campaign in the usual care arm and 53 (50.5%) are receiving the ARROW intervention. Hypothesis testing analysis, as well as cost-effectiveness and policy implication analysis, will be conducted. The 12-month end point results will be available in early 2026.

## Discussion

### Anticipated Findings

The ARROW program is an intervention designed to operate at 3 levels: the individual level, the health care provider level, and the health system level. At the individual level, we hypothesize that, compared to those receiving the one-time educational campaign, participants in the ARROW program will have increased uptake of early detection services and adherence to treatment, and this will, subsequently, improve health outcomes. At the health care provider level, we believe that the ARROW Provider Network will create an environment to link HIV pediatric and adult health care providers with their counterparts at the Cancer Diseases Hospital, which will facilitate peer-to-peer learning. Improved linkages between HIV and oncology care, as well as between pediatric and adult health care providers, will benefit both the health care providers and the AYA living with HIV who move through this health care landscape. At the health system level, the ARROW Health Care Collaborative will bring together health system administrators and Zambian Ministry of Health policy makers and foster an environment for collaborative problem solving. We believe that creating a communal space for health system level barriers will result in the successful implementation of solutions to address obstacles to care that AYA living with HIV experience.

### Limitations and Approaches to Minimize Bias

First, although cluster randomization reduces contamination across study arms, it increases the risk that the AYA living with HIV in each arm may differ at baseline. The study sampling frame, based on the HIV registers, will allow us to assess baseline differences before facility randomization and adjust our sampling process with propensity score weights. Second, data could be missing because of nonresponse and study attrition. The PCs will maintain regular contact with the AYA living with HIV, and we will use rigorous field data collection practices, including training data collectors, developing protocols, and monitoring fidelity continually. Furthermore, we will address any missing data by including demographic covariates that will serve as proxies for dropout and conducting sensitivity analyses. Third, we anticipate health care provider turnover at the HIV treatment facilities and will conduct refresher training sessions as needed throughout the trial period.

### Impact and Policy Implications

The model tested in Zambia can serve as a blueprint for other sub-Saharan African countries to ensure that AYA living with HIV receive optimal services across the cancer care continuum. We will host consultation and policy forums to share findings and seek feedback on the generalizability of the ARROW program to other sub-Saharan settings. The ARROW program, if shown to be successful, will provide a framework for implementing integrated HIV and cancer services to improve outcomes and reduce mortality among AYA living with HIV.

## Appendix

Multimedia Appendix 1 SPIRIT (Standard Protocol Items: Recommendations for Interventional Trials) checklist.

Multimedia Appendix 2 CONSORT (Consolidated Standards of Reporting Trials) checklist.

Multimedia Appendix 3 Peer review report from the ZCA1 SRB-K (M2) - National Cancer Institute Special Emphasis Panel - Cancer Control in People Living with HIV in LMIC, National Cancer Institute (National Institutes of Health, USA).

## Abbreviations

ARROW: AYAHIV Role-Based Responsibilities for Oncology-Focused Workforce
ART: antiretroviral therapy
AYA: adolescents and young adults
CC: cervical cancer
KS: Kaposi sarcoma
NCCN: National Comprehensive Cancer Network
NHL: non-Hodgkin lymphoma
PC: peer counselor

## Data Availability

Data sharing is not applicable to this article as no data sets were generated or analyzed during this study.
