# Supplementary material for: Integrated Model of Cancer Control for Early Detection and Treatment in Adolescents and Young Adults Living With HIV: Protocol for a Cluster Randomized Controlled Trial
Source: JMIR Res Protoc. 2025 Aug 29;14:e68254. doi: 10.2196/68254 (PMC12432473; doi:10.2196/68254)
Supplement: Multimedia Appendix 3 [file resprot_v14i1e68254_app3.pdf]

SUMMARY STATEMENT

(Privileged Communication)

PROGRAM CONTACT:
Vidya Vedham
240-276-7272
vidya.vedham@nih.gov

Release Date: 04/19/2022
Revised Date:

Application Number: 1 U01 CA275054-01

Principal Investigators (Listed Alphabetically):

MBIZVO, MICHAEL
MWABA, CATHERINE KASONGO
SUBRAMANIAN, SUJHA (Contact)

Applicant Organization: RESEARCH TRIANGLE INSTITUTE

Review Group: ZCA1 SRB-K (M2)
National Cancer Institute Special Emphasis Panel
Cancer Control in People Living with HIV in LMIC (U01)
AIDS - EXP. REV.

Meeting Date: 03/10/2022
Council: MAY 2022
Requested Start: 07/01/2022

RFA/PA: CA21-056
PCC: AEGH

Project Title: Integrated delivery of cancer control interventions for adolescents and young adults living with HIV in Zambia
SRG Action: Impact Score:23
Next Steps: Visit https://grants.nih.gov/grants/next_steps.htm
Human Subjects: 30-Human subjects involved - Certified, no SRG concerns
Animal Subjects: 10-No live vertebrate animals involved for competing appl.
Gender: 1A-Both genders, scientifically acceptable
Minority: 5A-Only foreign subjects, scientifically acceptable
Age: 1A-Children, Adults, Older Adults, scientifically acceptable

Project Year	Direct Costs Requested	Estimated Total Cost
1	387,563	782,346
2	332,588	671,372
3	385,065	777,303
4	385,571	778,325
5	440,489	889,184
TOTAL	1,931,276	3,898,529

1U01CA275054-01 SUBRAMANIAN, SUJHA

RESUME AND SUMMARY OF DISCUSSION: The goal of this multi-PI (MPI) U01 application, submitted in response to RFA-CA-21-056, is to develop and test an integrated model of cancer control for adolescents and young adults living with HIV (AYAHIV) that can be delivered as an embedded component in existing HIV treatment programs in primary care facilities and linked with specialist treatment in cancer centers. Specifically, the applicants will 1) Conduct randomized trials to compare the AYAHIV Role-based Responsibilities for Oncology-focused Workforce (ARROW) program with the one-time education campaign in increasing services received by AYAHIV to facilitate early diagnosis and in improving adherence to cancer treatment (Aim 1); 2) Use mixed methods to assess implementation outcomes of the ARROW program compared with one- time education based on acceptability, feasibility, appropriateness, fidelity, and sustainability (Aim 2) and finally 3) Perform economic evaluations to assess cost-effectiveness and return-on-investment scenarios (Aim 3). Strengths of this application include the focus on AYAHIV, a particularly vulnerable but steadily growing subset of PLWH with comparatively poorer outcomes than adult PLWH; the focus on improving adherence to cancer treatment among AYAHIV with malignant diagnoses and reducing abandonment of treatment; the well-rounded team of clinicians, implementation scientists, economists, administrators, and data analysts; well-described MPI plan; the novel design that investigates the facilitation of early diagnosis as well as the receipt of optimal treatment, targeting both HIV clinics and cancer centers; the multi-level ARROW strategy informed by implementation science theory; well-described program, which will be developed with input, testing, and adaption across all types of participants involved; detailed and comprehensive statistical plan and power analysis; carefully designed and rigorous cost effectiveness analysis and the strong environment at Research Triangle Park and infrastructure in Zambia. However, there is concern about the cancer adherence part of Aim 1b. It is unclear if there will be contamination between those participating in cancer screening portion and whether they will be excluded or not. Nonetheless, successful execution of the aims of this application will yield a set of data-driven strategies that can be scaled up to reduce premature cancer mortality among AYAHIV. Therefore, the overall impact of this application is anticipated to be high.

DESCRIPTION (provided by applicant): Zambia is a global epicenter for HIV with a large number of adolescents and young adults living with HIV (AYAHIV). Compared with their uninfected peers, AYAHIV are at increased risk of developing cancer, most frequently Kaposi sarcoma (KS), non-Hodgkin's lymphoma (NHL), and cervical cancer (CC). To reduce this premature mortality, evidence-based strategies should be implemented to both diagnose cancers at an earlier stage and help AYAHIV complete recommended cancer treatment. We will use theory-informed multilevel strategies to create the AYAHIV Role-based Responsibilities for Oncology-focused Workforce (ARROW) program to increase uptake of services for early diagnosis and improve compliance with cancer treatment for KS, NHL, and CC. Our overall approach is based on the evidence-based strategy of peer support for engagement and learning. At the individual level, we will address barriers by embedding peer counselors to support AYAHIV. At the provider level, we will create a peer-to-peer learning network to build linkages between those specializing in pediatric and adult HIV treatment and cancer care. At the health care system level, we will bring together health care administrators and Zambian Ministry of Health policy makers to review barriers and to develop and implement collaborative solutions. We will use implementation science methods to evaluate effectiveness, implementation outcomes, and cost-effectiveness of the ARROW program compared with a one-time education campaign by pursuing the following aims: Aim 1. Conduct randomized trials to compare the ARROW program with the one-time education campaign in increasing services received by AYAHIV to facilitate early diagnosis (physical exam for KS and NHL, CC screening, and timely diagnostic testing) and in improving adherence to cancer treatment. Aim 2. Use mixed methods to assess implementation outcomes of the ARROW program compared with one- time education based on acceptability, feasibility, appropriateness, fidelity, and sustainability. Aim 3. Perform economic evaluations to assess cost-effectiveness and return-on-investment scenarios. Successful completion of these aims will yield a set of data-driven strategies that

can be scaled up to reduce premature cancer mortality among AYAHIV. To support future implementation efforts, we will generate incremental cost-effectiveness estimates, conduct policy simulations, evaluate implementation outcomes, and assess challenges and facilitators to optimize the ARROW program. The model tested in Zambia can serve as a blueprint for other Sub-Saharan African countries to ensure AYAHIV receive optimal services to facilitate early diagnosis and ensure completion of guideline-recommended treatments. The ARROW program will also provide a framework for implementing expanded services, such as preventive services and survivorship care, to further reduce the burden of cancer AYAHIV face.

PUBLIC HEALTH RELEVANCE: Adolescents and young adults living with HIV experience higher premature mortality from cancer than their uninfected peers. In Zambia, because of multilevel barriers, interventions for early diagnosis and optimized treatment to reduce this cancer mortality are underutilized for this disproportionally impacted cohort. In this study, we will test peer-to-peer education and support strategies to increase use of early diagnosis services in HIV treatment facilities and improve compliance with cancer treatment in the cancer center.

CRITIQUE: The written critiques of individual reviewers are provided in essentially unedited form in this section. Please note that critiques and criteria scores, prepared prior to the review meeting, may not have been revised following discussions at the meeting. The "Resume and Summary of Discussion" section summarizes the final opinions of the review committee.

CRITIQUE 1

Significance:	1
Investigator(s):	1
Innovation:	1
Approach:	4
Environment:	1

Overall Impact: This is an application on integrated delivery of cancer control interventions for adolescents and young adults living with HIV in Zambia. Goal is to create a program to increase uptake of services for early diagnosis and improve compliance with cancer treatment for Kaposi Sarcoma (KS), non-Hodgkin's lymphoma (NHL), and cervical cancer (CC). This will be done using theory-informed multi-level strategies to create the ARROW program, using evidence-based strategy of peer support for engagement and learning using peer counselors for individual participants and peer-to-peer learning network for providers. The first aim is a randomized trial, second aim is mixed methods to look at implementation-specific outcomes, and third is a cost effectiveness analysis. And also addresses systemic barriers by bringing in policy makers and heath administrators to implement collaborative solutions. Strategies are guided by COM-B model (capability, opportunity, and motivation). Content will be AYA focused. Issue is not just early detection but also early intervention, which happens in separate clinics. Dr. Subramanian (PI) is a health economist from RTI with numerous experiences looking at care delivery for early diagnoses and treatment of disease. Has an ongoing and recently completed NIH projects in Zambia for HIV prevention and treatment as well as cervical cancer prevention strategies. Dr. Catherine Mwaba (MPI) is head of Clinical and Radiation Oncology at Cancer Diseases Hospital in Zambia. Team has experience in Zambia as well as collaborators at RTI and other locations on other projects with the PI, seem well suited for the application. Early diagnosis cluster randomized trial is adequately powered and designed. The second part that focuses on adherence to cancer treatment, is a little less clear. it sounds like participants are randomly allocated to ARROW or education, but could they already be exposed to ARROW depending on the screening clinic they participated in? So, there could be contamination there. Or are the participants in the screening and treatment trials unique and not able to overlap? It is unclear why the clustering is not still a factor for treatment adherence? Won't

some providers be part of ARROW and some won't, so the clustering should be by provider? it is a cluster randomized trial so wouldn't that depend on where they go for care? It sounds like a cluster randomized trial for both, but the stats do not align to cancer treatment (which reads more like individual randomization). Needs to account for ICC and the fact that providers will be trained at these clinics therefore approach needs to be consistent across the clinic level.

1. Significance:
Strengths
- Interventions for early diagnosis and optimized treatment of AYAHIV cancers are underutilized in Zambia.
- Need to focus on tailoring to AYAHIV.
- Team has experience working with AYAHIV and cancer and designing AYA friendly programs.
- AYAHIV is an at-risk population, with poor outcomes and barriers to care, including transitioning from pediatric to adult clinics, stigma, and no link between primary care HIV providers and cancer specialists.

Weaknesses
- None noted.

2. Investigator(s):
Strengths
- Very strong team. Has experience in Zambia and on projects with AYAHIV specific to CC, NHL, and KS.
- RTI will handle data/analyses and economic considerations and are well equipped to do so.
- Have well-rounded team of clinicians, implementation scientists, economists, administrators, and data analysts.

Weaknesses
- None noted.

3. Innovation:
Strengths
- Will incorporate feedback from AYAHIV in development of program.
- Approach tests both early diagnosis as well as receipt of optimal treatment to show continuity of care.
- Program targets multiple cancer types and could be expanded in the future.

Weaknesses
- None noted.

4. Approach:
Strengths
- Strong outline of how the program will be developed, with input, testing, and adaption across all types of participants involved.
- Will train peer counselors with well-described process.
- Strong plan for providers with training.
- Early diagnosis Aim 1a is cluster randomized with 18 clinics, stratified by regional population. 2 primary objectives, split alpha, both adequately powered and looking for 20% improvement in rates. ICC in range 0.03-0.05 (fairly standard for cluster randomized trials).
- Aims 2 and 3 are well outlined with appropriate analysis plans.

Weaknesses
- Concerned about the cancer adherence part of Aim 1b. Unclear if there will be contamination between those participating in cancer screening portion and whether they will be excluded or not. And if there is provider training in this piece as well, it might need to be clustered by provider seen at CDH. It is just unclear and needs some more justification.

5. Environment:
Strengths
- Very strong environment and collaboration between established clinics in Zambia and DCC in RTI.

Weaknesses
- None noted.

Study Timeline:
Strengths
- Clearly outlined with deliverables in each year.

Weaknesses
- None noted.

Protections for Human Subjects
Acceptable Risks and/or Adequate Protections.
- Well described with risks and benefits.

Data and Safety Monitoring Plan (Applicable for Clinical Trials Only):
Acceptable.

Inclusion Plans (Applicable Only for Human Subjects research and not IRB Exemption #4)
- Sex/Gender: Distribution justified scientifically.
- Race/Ethnicity: Distribution justified scientifically.
- Inclusion/Exclusion Based on Age: Distribution justified scientifically.
- Statistical design and power section is a bit sparse compared to the grant itself. But analytic plans, outcomes, etc. are well described for all aims in the main body of the grant.

Vertebrate Animals
Not Applicable (No Vertebrate Animals).

Biohazards
Not Applicable (No Biohazards).

Applications from Foreign Organizations
Justified.
- Appropriate as trial will be done in Zambia.

Select Agents
Not Applicable (No Select Agents).

Resource Sharing Plans
Acceptable.

Authentication of Key Biological and/or Chemical Resources
Not Applicable (No Relevant Resources).

Budget and Period of Support
Recommend as Requested.

CRITIQUE 2

Significance: 2
Investigator(s): 2
Innovation: 2
Approach: 2
Environment: 2

Overall Impact: This MPI application is designed to test the implementation of integrated delivery of cancer control interventions for AYALWH in Zambia, with the development of the ARROW (AYAHIV Role-based Responsibilities for Oncology-focused Workforce) program, with the goal of increasing the update of services for early diagnosis and to improve compliance with cancer treatment for Kaposi Sarcoma, Non-Hodgkin Lymphoma and Cervical Cancer. The intervention includes a randomized trial. The investigators and research environment are outstanding. The design is novel and could result in significant advances in the development of integrated models for delivery of cancer control strategies for multiple cancers. Impact potential is high.

1. Significance:
The application focuses on a vulnerable population (AYALWH) with documented premature mortality due to cancer in need of focused interventions that address multilevel barriers. Interventions for early diagnosis and optimized treatment of AYALWH cancer are underutilized. The ARROW program includes a randomized clinical trial to address both early diagnosis and cancer treatment adherence that can result in the development of a low-cost, multilevel peer to peer support and learning strategies that could be scalable and exported to other LMICs, particularly in SSA where the prevalence of HIV is the highest. The application is based on a theoretical framework and follows and socioecological model, with the potential result of strategies that target barriers at the individual, providers, and health systems levels.
Strengths
- Comprehensive and innovative study design that targets multi-level interventions.
- Addresses the three most common cancer diagnoses in AYALWH.
- Randomized clinical trial design.
- Leverages existing infrastructure and systems for HIV diagnosis and treatment.

Weaknesses
- None noted.

2. Investigator(s):
Three Principal Investigators that combine significant expertise and with a long history of research collaborations in the field. Dr. Subramanian is a health economist with research experience in Zambia where has led the development of studies delivering targeted prevention and screening for women and adolescents with HIV. She has a very strong track record of peer-reviewed federal funding and is currently working with Population Council (Zambia) to implement a multi-level intervention to increase HIV testing and ATR adherence among AYALWH. She also has a strong publication track record on implementation science to improve care for AYALWH, integrated delivery of health services for cancer and NCDs, and development of methods for cost-effective interventions. Dr. Mwaba is the head of oncology at Cancer Diseases Hospital in Lusaka, and the leader and founder of ZASCRO. He has a good track record of research collaborations with international partners and has published on the care for HIV-related malignancies. Dr. Mbizvo is the country director for the Population Council of Zambia and has worked previously with UN agencies and WHO, where he was the director of reproductive health and research. He also has experience in leading large teams in HIV research programs. The team of investigators includes behavioral and epidemiological scientists and experts in stigma and discrimination.
Strengths
- Representation of the continuum of research methodologies in implementation sciences, health economics and behavioral sciences.

- Very strong engagement and empowerment of Zambian investigators that will facilitate research capacity building necessary for scale-up.

Weaknesses
- None noted.

3. Innovation:
This application includes a comprehensive three-step sequential approach that employs a user-centered design to ensure age-appropriate messaging and services for AYALWH. The design is novel in that it investigates the facilitation of early diagnosis as well as the receipt of optimal treatment, targeting both HIC clinics and cancer centers. The ARROW program uses a modular process.
Strengths
- Randomized approach that targets both early diagnosis and adherence to treatment.
- The study has the potential to influence care for multiple cancers.
- The study has the potential to integrate HIV and cancer centers through a multi-level intervention approach.

Weaknesses
- None noted.

4. Approach:
The approach includes three phases (pre-implementation, intervention and interim assessments, and final analyses, including economic evaluations) and includes a community advisory board and a youth advisory board. Aim 1 is a randomized trial that compares the ARROW intervention to the standard, both for early diagnosis as well as adherence to treatment. Aim 2 includes mixed methods to assess the implementation outcomes at the patients, providers, and health care systems. Aim 3 includes cost effectiveness and return of investment methods for evaluate outcomes and impact.
Strengths
- Comprehensive design that is cancer type-agnostic.
- Targets early diagnosis and cancer treatment adherence.
- Strong involvement of local investigators.

Weaknesses
- Integration of mobile technology could have been considered in the study design.

5. Environment:
The investigators at RTI and Zambia are very complementary and bring a broad spectrum of research expertise, and the research team includes two MoH staff members as advisors. Importantly, the investigators have created equitable partnerships across institutions. There is strong record of collaboration between RTI and Population Council and ZASCRO in studies addressing AYALWH and cancer, research in workforce training, and in designing AYA-friendly programs. The study setting in Zambia includes HIV treatment clinics in urban, peri-urban, and rural populations and has established a very rich provider network and a healthcare collaborative, which includes six healthcare administrators and four MoH policymakers.
Strengths
- Strong track record of collaborations between partner institutions.
- Well balanced and equitable distribution of efforts and engagement.
- Builds strongly on existing research infrastructure and talent in Zambia.
- Strong commitment and political will by policymakers and administrators.

Weaknesses
- None noted.

Study Timeline:
Strengths
- None noted.

Weaknesses
- None noted.

Protections for Human Subjects
Acceptable Risks and/or Adequate Protections.
Data and Safety Monitoring Plan (Applicable for Clinical Trials Only):
Acceptable.

Inclusion Plans (Applicable Only for Human Subjects research and not IRB Exemption #4)
- Sex/Gender: Distribution justified scientifically.
- Race/Ethnicity: Distribution justified scientifically.
- Inclusion/Exclusion Based on Age: Distribution justified scientifically.

Vertebrate Animals
Not Applicable (No Vertebrate Animals).

Biohazards
Not Applicable (No Biohazards).

Applications from Foreign Organizations
Justified.

Select Agents
Not Applicable (No Select Agents).

Resource Sharing Plans
Not Applicable (No Relevant Resources).

Authentication of Key Biological and/or Chemical Resources
Not Applicable (No Relevant Resources).

Budget and Period of Support
Recommend as Requested.

CRITIQUE 3

Significance:	2
Investigator(s):	1
Innovation:	3
Approach:	2
Environment:	3

Overall Impact: In this well written and carefully designed application Dr. Subramaniam and colleagues define and propose a multilevel strategy for improving cancer screening in Adolescents and Young Adults with HIV (AYAHIV) in Zambia and improving the compliance with cancer treatment of Zambian AYAHIV who have been diagnosed with cancer. AYAHIV represent a growing proportion of PLWH globally; however, AYAHIV represent one of the most vulnerable subsets of PLWH because they demonstrate the poorest adherence to stable, viral-suppressive therapy with combination antiretrovirals. They face an increased risk of cancer compared with their HIV-negative peers, particularly an increased risk of the AIDS-defining cancers Kaposi sarcoma, non-Hodgkin lymphoma, and cervical cancer. Dr. Subramaniam and colleagues note that data on malignancies in AYAHIV in

sub-Saharan Africa are sparse, but late-stage presentation, unplanned breaks from cancer treatment, and abandonment of cancer treatment have been reported for this subset of PLWH, resulting in high morbidity and premature mortality. The objective of this application is to define and test an integrated model for reducing cancer morbidity and mortality amongst AYAHIV in Zambia that can be delivered as an embedded component in existing HIV treatment programs in primary care facilities and linked with specialist treatment in cancer centers. They have titled this approach the "**A**YAHIV **R**ole-based **R**esponsibilities for **O**ncology-Focused **W**orkforce," or "ARROW." ARROW is described as a multi-level strategy informed by implementation science theory that aims to increase uptake of services for early diagnosis and improve compliance with treatment for KS, non-Hodgkin lymphoma, and cervical cancer. It is based on the strategy of peer support for engagement and learning and has components that are intended to be applied at the level of individual patients, at the level of providers, physicians, and nurses, and at the health-care system level. In this application the investigative team proposes to implement the ARROW strategy and then to compare it with a one-time education campaign that gives informational brochures to AYAHIV and HIV providers. An attractive feature of this application is the rigorous cost-effectiveness analysis and return on investment analysis that are proposed in specific aim 3, particularly since it is readily anticipated that the ARROW program will prove superior to the one-time education campaign-based approach – knowing how much the anticipated improvements will cost is both important and valuable. If successful, the studies and activities proposed in this application could have a potentially large impact on cancer control in AYAHIV in Zambia. Moreover, the ARROW program tested in Zambia could serve as a blueprint for cancer control programs for AYAHIV in other sub-Saharan countries.

1. Significance:
Strengths
- Focus on AYAHIV, a particularly vulnerable but steadily growing subset of PLWH with comparatively poorer outcomes than adult PLWH.
- Focus on improving adherence to cancer treatment among AYAHIV with malignant diagnoses and reducing abandonment of treatment.

Weaknesses
- None noted.

2. Investigator(s):
Strengths
- The investigative team combining members from RTI, ZASCRO and the Cancer Diseases Hospital, and the Population Council have the requisite training, expertise, and experience to carry out the proposed studies. Dr. Subramaniam as contact PI among the MPIs will assume overall responsibility for management and fiscal oversight of the U01 award; Dr. Subramaniam has an excellent track record for management of large multi-year research awards involving large investigative teams.
- Drs. Subramaniam, Mwaba, and Mbizvo from RTI, ZASCRO, and the Population Council, respectively, will serve as MPIs for this application; the roles and responsibilities of each MPI is clearly articulated in the application.

Weaknesses
- None noted.

3. Innovation:
Strengths
- Focus on AYAHIV as a particularly vulnerable subset within PLWH.

Weaknesses
- None noted.

4. Approach:

Strengths
- Design of the multi-level ARROW strategy informed by implementation science theory.
- Detailed and comprehensive statistical plan and power analysis.
- Detailed, carefully designed cost effectiveness analysis.

Weaknesses
- Focus on the AIDS-defining cancers KS, NHL, and cervical carcinoma; as more is learned about the natural history of HIV infection in AYA, and more data on cancer incidence in AYAHIV becomes available, the number of different malignancies for which AYAHIV may be at increased risk may well rise.

5. Environment:
Strengths
- The scientific environments at RTI, ZASCRO / Cancer Diseases Hospital, and the Population Council are individually excellent.
- The ARROW study activities will be conducted in large part at selected HIV treatment clinics in Lusaka Province that serve at least 400 AYAHIV and at the Cancer Diseases Hospital; from the descriptions provided in the application, these facilities sound well suited to the proposed studies.

Weaknesses
- None noted.

Study Timeline:
Strengths
- Very carefully thought out and detailed; seems realistic.

Weaknesses
- None noted.

Protections for Human Subjects
Acceptable Risks and/or Adequate Protections.
Data and Safety Monitoring Plan (Applicable for Clinical Trials Only):
Acceptable.

Inclusion Plans (Applicable Only for Human Subjects research and not IRB Exemption #4)
- Sex/Gender: Distribution justified scientifically.
- Race/Ethnicity: Distribution justified scientifically.
- Inclusion/Exclusion Based on Age: Distribution justified scientifically.

Vertebrate Animals
Not Applicable (No Vertebrate Animals).

Biohazards
Not Applicable (No Biohazards).

Applications from Foreign Organizations
Justified.

Select Agents
Not Applicable (No Select Agents).

Resource Sharing Plans
Not Applicable (No Relevant Resources).

Authentication of Key Biological and/or Chemical Resources
Not Applicable (No Relevant Resources).

Budget and Period of Support
Recommend as Requested.

THE FOLLOWING SECTIONS WERE PREPARED BY THE SCIENTIFIC REVIEW OFFICER TO SUMMARIZE THE OUTCOME OF DISCUSSIONS OF THE REVIEW COMMITTEE, OR REVIEWERS' WRITTEN CRITIQUES, ON THE FOLLOWING ISSUES:

PROTECTION OF HUMAN SUBJECTS: ACCEPTABLE

INCLUSION OF WOMEN PLAN: ACCEPTABLE

INCLUSION OF MINORITIES PLAN: ACCEPTABLE

INCLUSION ACROSS THE LIFESPAN: ACCEPTABLE

COMMITTEE BUDGET RECOMMENDATIONS: The budget was recommended as requested.

Footnotes for 1 U01 CA275054-01; PI Name: SUBRAMANIAN, SUJHA

NIH has modified its policy regarding the receipt of resubmissions (amended applications).See Guide Notice NOT-OD-18-197 at https://grants.nih.gov/grants/guide/notice-files/NOT-OD-18-197.html. The impact/priority score is calculated after discussion of an application by averaging the overall scores (1-9) given by all voting reviewers on the committee and multiplying by 10. The criterion scores are submitted prior to the meeting by the individual reviewers assigned to an application, and are not discussed specifically at the review meeting or calculated into the overall impact score. Some applications also receive a percentile ranking. For details on the review process, see http://grants.nih.gov/grants/peer_review_process.htm#scoring.

MEETING ROSTER

National Cancer Institute Special Emphasis Panel
NATIONAL CANCER INSTITUTE
Cancer Control in People Living with HIV in LMIC (U01)

ZCA1 SRB-K (M2)
03/10/2022

Notice of NIH Policy to All Applicants: Meeting rosters are provided for information purposes only. Applicant investigators and institutional officials must not communicate directly with study section members about an application before or after the review. Failure to observe this policy will create a serious breach of integrity in the peer review process, and may lead to actions outlined in NOT-OD-14-073 at https://grants.nih.gov/grants/guide/notice-files/NOT-OD-14-073.html, NOT-OD-15-106 at https://grants.nih.gov/grants/guide/notice-files/NOT-OD-15-106.html, and NOT-OD-18-115 at https://grants.nih.gov/grants/guide/notice-files/NOT-OD-18-115.html, including removal of the application from immediate review.

CHAIRPERSON(S)

BEYRER, CHRISTOPHER, MD, MPH
PROFESSOR, DESMOND M. TUTU PROFESSOR IN PUBLIC HEALTH AND HUMAN RIGHTS
DEPARTMENT OF EPIDEMIOLOGY, NURSING AND MEDICINE
BLOOMBERG SCHOOL OF PUBLIC HEALTH
JOHNS HOPKINS UNIVERSITY
BALTIMORE, MD 21205

CHAMPION, VICTORIA LEE, PHD
PROFESSOR
DEPARTMENT OF POPULATION SCIENCE RESEARCH
ASSOCIATE DIRECTOR OF CANCER CONTROL
AND POPULATION SCIENCES IUSCC
INDIANA UNIV-PURDUE UNIV AT INDIANAPOLIS
INDIANAPOLIS, IN 46202

MEMBERS

ABU-RMEILEH, NIVEEN, PHD
PROFESSOR
DEPARTMENT OF EPIDEMIOLOGY AND PUBLIC HEALTH
INSTITUTE OF COMMUNITY AND PUBLIC HEALTH
BIRZEIT UNIVERSITY
JERUSALEM 973000
PALESTINIAN TR

ASGARY, RAMIN, MD, MPH
ASSOCIATE PROFESSOR
DEPARTMENT OF GLOBAL HEALTH
SCHOOL OF PUBLIC HEALTH
GEORGE WASHINGTON UNIVERSITY
WASHINGTON, DC 20052

BOFFETTA, PAOLO, MPH, MD
PROFESSOR
DEPARTMENT OF FAMILY, POPULATION AND PREVENTATIVE MEDICINE
RENAISSANCE SCHOOL OF MEDICINE
STATE UNIVERSITY OF NEW YORK STONY BROOK
STONY BROOK, NY 11794

CATTAMANCHI, ADITHYA, MD, MAS
PROFESSOR
DEPARTMENT OF EPIDEMIOLOGY AND BIOSTATISTICS
DIVISION OF PULMONARY AND CRITICAL CARE MEDICINE
SAN FRANCISCO GENERAL HOSPITAL
UNIVERSITY OF CALIFORNIA, SAN FRANCISCO
SAN FRANCISCO, CA 94110

DRESSLER, EMILY VAN METER, PHD
ASSOCIATE PROFESSOR
DEPARTMENT OF BIOSTATISTICS AND DATA SCIENCE
WAKE FOREST SCHOOL OF MEDICINE
WINSTON-SALEM, NC 27157

GENG, ELVIN H., MD, MPH
PROFESSOR
DEPARTMENT OF INTERNAL MEDICINE
DIVISION OF INFECTIOUS DISEASES
WASHINGTON UNIVERSITY
ST. LOUIS, MO 63110

JOHN-STEWART, GRACE, MD, PHD, MPH
PROFESSOR
DEPARTMENTS OF GLOBAL HEALTH, EPIDEMIOLOGY, MEDICINE, AND PEDIATRICS
UNIVERSITY OF WASHINGTON
SEATTLE, WA 98104

LAJOUS, MARTIN, SCD, MD
ASSOCIATE PROFESSOR
DEPARTMENT OF EPIDEMIOLOGY
MEXICO NATIONAL INSTITUTE OF PUBLIC HEALTH
MEXICO CITY 14000
MEXICO

MORSE, GENE D., PHARMD, BCPS, FCCP
DISTINGUISHED PROFESSOR AND DIRECTOR
DEPARTMENT OF PHARMACY PRACTICE
CENTER FOR INTEGRATED GLOBAL BIOMEDICAL SCIENCES
SUNY GLOBAL HEALTH INSTITUTE
UNIVERSITY AT BUFFALO
BUFFALO, NY 14203

PAZ-SOLDAN, VALERIE ANDREA, PHD, MPH
ASSOCIATE PROFESSOR, DIRECTOR OF TULANE HEALTH OFFICES FOR LATIN AMERICA
DEPARTMENT OF TROPICAL MEDICINE
SCHOOL OF PUBLIC HEALTH AND TROPICAL MEDICINE
TULANE UNIVERSITY
NEW ORLEANS, LA 70112

RANDALL, THOMAS C., MD
ASSOCIATE PROFESSOR; DIRECTOR, GLOBAL ONCOLOGY INITIATIVE
DEPARTMENT OF OBSTETRICS AND GYNECOLOGY
HARVARD CANCER CENTER
MASSACHUSETTS GENERAL HOSPITAL
BOSTON, MA 02108

RODRIGUEZ-GALINDO, CARLOS, MD
CHAIR AND EXECUTIVE VICE-PRESIDENT
DEPARTMENT OF GLOBAL PEDIATRIC MEDICINE
ST. JUDE CHILDREN'S RESEARCH HOSPITAL
MEMPHIS, TN 38105

VAN LOON, KATHERINE, MD, MPH
ASSOCIATE PROFESSOR OF CLINICAL MEDICINE
DIVISION OF HEMATOLOGY/ONCOLOGY
DIRECTOR, GLOBAL CANCER PROGRAM
HELEN DILLER FAMILY COMPREHENSIVE CANCER CENTER
UNIVERSITY OF CALIFORNIA, SAN FRANCISCO
SAN FRANCISCO, CA 94143

WARREN, EDUS HOUSTON, MD, PHD
PROFESSOR
DIVISIONS OF CLINICAL RESEARCH AND
VACCINE AND INFECTIOUS DISEASE
PROGRAM HEAD, GLOBAL ONCOLOGY
FRED HUTCHINSON CANCER RESEARCH CENTER
SEATTLE, WA 98109

SCIENTIFIC REVIEW OFFICER

SIDDIQUI, HASAN, PHD
SCIENTIFIC REVIEW OFFICER
SPECIAL REVIEW BRANCH
DIVISION OF EXTRAMURAL ACTIVITES
NATIONAL CANCER INSTITUTE
NATIONAL INSTITUTES OF HEALTH
ROCKVILLE, MD 20850

EXTRAMURAL SUPPORT ASSISTANT

LEE, JULIA Y.
STAFF ASSISTANT
SPECIAL REVIEW BRANCH
DIVISION OF EXTRAMURAL ACTIVITIES
NATIONAL CANCER INSTITUTE SHADY GROVE
NATIONAL INSTITUTES OF HEALTH
ROCKVILLE, MD 20850

Consultants are required to absent themselves from the room during the review of any application if their presence would constitute or appear to constitute a conflict of interest.
